# Association between endothelial function and early neurological improvement in atrial fibrillation-related ischemic stroke

**DOI:** 10.3389/fneur.2026.1733034

**Published:** 2026-01-29

**Authors:** So Young Yang, Sung Hee Ahn, Jeonggeun Moon, Yeong-Bae Lee, Dae-il Chang, Sang Hee Ha

**Affiliations:** 1 Department of Cardiology, Kyung Hee University College of Medicine, Kyung Hee University Hospital at Gangdong, Seoul, Republic of Korea; 2 Department of Cardiology, Gil Medical Center, Gacheon University, Incheon, Republic of Korea; 3 Department of Big Data Management and Engineering, Namseoul University, Chungcheongnam-do, Republic of Korea; 4 Department of Neurology, Gil Medical Center, Gacheon University, Incheon, Republic of Korea

**Keywords:** atrial fibrillation, atrial fibrillation stroke, early neurologic improvement, endothelial function, flow-mediated dilation

## Abstract

**Background:**

Understanding the factors related to early neurological improvement (ENI) is crucial in managing atrial fibrillation-related ischemic stroke (AF-stroke), as ENI indicates better long-term outcomes. We investigated the association between endothelial function, measured via flow-mediated dilation (FMD), and the occurrence of ENI in patients with AF-stroke.

**Methods:**

We reviewed patients with acute AF-stroke within 7 days of FMD between April 2019 and April 2025. ENI was defined as a ≥2-point decrease in National Institutes of Health Stroke Scale (NIHSS) or ≥1-point reduction in motor NIHSS items within 24 h in non-thrombolysis patients. For thrombolysis patients, ENI was a ≥8-point reduction or NIHSS 0–1 at 24 h. FMD was measured during hospitalization and expressed as %FMD = (peak diameter − baseline diameter) / Baseline diameter × 100. Multivariable analysis identified the factors associated with ENI and explored their relationship with FMD.

**Results:**

Among the 169 patients diagnosed with AF-stroke, 77 (44.4%) experienced ENI. Those with ENI had higher NIHSS (7 [4–13] vs. 2 [1–5], *p* < 0.001), more confluent (38.7% vs. 25.5%) and scattered with confluent pattern (29.3% vs. 18.1%, *p* = 0.007), and higher %FMD (6.5% ± 2.5% vs. 5.3 ± 2.2%, *p* = 0.001). Multivariable analysis revealed a higher initial NIHSS score (adjusted odds ratio [aOR]: 1.329, *p* < 0.001) and a history of smoking (aOR: 4.532, *p* = 0.004), and higher %FMD score (aOR: 1.179; *p* = 0.043) were independently associated with ENI. Subgroup analysis demonstrated a stronger association between high %FMD and ENI in patients with concomitant vascular risk factors, such as hypertension and dyslipidemia.

**Conclusion:**

Endothelial function was associated with ENI in patients with AF-stroke.

## 1 Introduction

More than 20% of stroke cases are caused by cardiac embolism attributable to atrial fibrillation (AF) (1). Compared to strokes of other etiologies, AF-related ischemic stroke (AF-stroke) is associated with greater severity and a higher risk of disability and mortality (2). This may be due to large infarct volumes resulting from the abrupt occlusion of blood flow by a clot originating in the heart, often without sufficient time for collateral circulation to compensate (3). Numerous studies have shown that AF-stroke is associated with a lower likelihood of early neurological improvement (ENI) (2, 4, 5). Nevertheless, ENI can still occur in a subset of patients with AF-stroke and is associated with favorable functional outcomes (6).

Several factors have been linked to ENI, including successful recanalization, collateral status, and smaller infarct core size (6–8). Additionally, the endothelium plays multiple roles in stroke pathophysiology and recovery by regulating vascular tone, maintaining blood–brain barrier (BBB) integrity, modulating inflammatory responses, and influencing thrombotic and fibrinolytic balance (9–11). Patients who have experienced acute ischemic stroke (AIS) with impaired FMD are associated with early neurological deterioration and poor long-term outcome (12). Furthermore, endothelial function is associated with the development of AF and AF-stroke by promoting atrial remodeling, thrombogenesis, and hemodynamic alterations (13, 14). However, whether endothelial function influences the capacity for early neurological recovery in patients with AF-stroke remains unclear.

Brachial artery flow-mediated dilation (FMD), assessed using high-resolution ultrasonography, is a widely used tool to evaluate endothelial function (15). In this study, we aimed to identify the factors associated with ENI in patients with AF-stroke, with a particular focus on the role of endothelial function as measured by FMD.

## 2 Materials and methods

### 2.1 Participants

We retrospectively reviewed the data of patients with acute AF-stroke (within 7 days of stroke onset) who were admitted to Gil Medical Center between April 2019 and April 2025. We included patients with known or newly diagnosed AF during hospitalization and with diffusion-weighted imaging-positive lesions. Additionally, patients underwent FMD assessment during hospitalization when they were neurologically stable.

We excluded patients who met any of the following criteria: (1) poor initial magnetic resonance imaging (MRI) quality; (2) incomplete clinical data; (3) a significant (> 50%) stenosis of the corresponding artery; (4) clinical syndrome typical of small-vessel disease (SVD) associated with a subcortical lesion with maximal diameter < 15 mm; (5) presence of other causes, such as dissection, vasculitis, cancer-related stroke; (6) undetermined causes such as two or more mechanisms (i.e., SVD or large artery atherosclerosis combined with AF-stroke) or negative; and (7) inability to reliably undergo high-resolution ultrasonography due to lack of cooperation. This study was approved by the local ethics committee of Gil Medical Center, South Korea (GAIRB number: 2023-119).

### 2.2 Clinical data and ENI

Clinical data and risk factors were obtained from the patients’ medical records and the stroke registry database. The use of previous medications, including antithrombotics and statins, were also assessed. Neurological deficits due to stroke were evaluated at admission using the National Institutes of Health Stroke Scale (NIHSS) score.

The time of stroke onset was estimated from the first abnormal finding in patients with clear stroke onset. If patients were eligible for IV alteplase treatment, they underwent endovascular thrombectomy (EVT) after receiving a standard dose of IV alteplase (0.9 mg/kg of body weight; 10% administered as a bolus followed by 1-h infusion of the remaining dose). All patients underwent EVT in accordance with national clinical practice guidelines and local protocols. Electrocardiograms were obtained from the emergency department and monitored during admission to the stroke units. Transthoracic echocardiography (TTE) was performed in all patients during admission. Left ventricular ejection fraction (LVEF) was measured using the biplane Simpson method. The left atrial volume index (LAVI) was calculated using the biplane area–length method and indexed to the body surface area. Newly documented AF cases were also evaluated.

ENI was defined as a ≥2-point decrease in NIHSS or ≥ 1-point reduction in motor NIHSS items within 24 h in non-thrombolysis patients (16). For thrombolysis patients, ENI was defined as an 8-point reduction or an NIHSS score of 0–1 at 24 h (17).

### 2.3 Imaging assessment

All patients underwent MRI using a 3.0 T Philips scanner (Philips Healthcare, Eindhoven, Netherlands). Stroke lesion patterns were categorized as multiple scattered, single confluent, and multiple with confluent lesions. Multiple scattered lesions were defined as multiple small ischemic lesions of < 15 mm in diameter. Multiple with confluent lesions were defined by the presence of lesions ≥ 15 mm along with additional lesions of any size in any arterial territory. Single confluent lesion was defined as a single lesion of ≥15 mm (18). The lesion location was categorized as anterior, posterior, or both.

### 2.4 Flow-mediated dilation

During admission, endothelial function was assessed by measuring the FMD of the brachial artery on the non-paretic arm in response to hyperemia. This was performed using high-resolution B-mode ultrasound (Aplio 50 Toshiba SSA-700) with a 7.5 MHz linear array transducer. Measurements were performed once the patients were neurologically stable. The participants were asked to abstain from food and caffeine for 12 h before the ultrasound scan, and no medication was administered from midnight until after the procedure.

The brachial artery was located longitudinally at a standardized point approximately 2–3 cm above the antecubital fossa. The baseline diameter and blood flow velocity were recorded continuously for 10 s. A blood pressure cuff was placed on the forearm and inflated to at least 50 mmHg above the participant’s systolic pressure for 5 min to induce occlusion. Twenty seconds before the cuff deflation, the diameter and blood flow velocity were recorded. After 5 min, the cuff was deflated to allow for reactive hyperemia in the hand and forearm, which led to vasodilation of the brachial artery. The percentage of FMD was calculated as 100 × [(peak diameter/baseline diameter)/ baseline diameter] (19).

### 2.5 Statistical analysis

The baseline characteristics were compared between the ENI and non-ENI groups. Continuous variables were expressed as the mean ± SD or median (IQR); categorical variables as frequencies and percentages. Group comparisons were performed using *t*-tests, Mann–Whitney U tests, or chi-square tests, as appropriate. In addition, the patients’ baseline characteristics were compared according to the tertiles of% FMD. The chi-square test (Fisher’s exact test) was used for categorical variables and analysis of variance (ANOVA) was used for continuous variables. ANOVA and the Kruskal–Wallis test were used to determine significant differences. The predictors of ENI were identified using logistic regression analysis. Variables with *p* < 0.1 in the univariable analysis, along with clinically relevant covariates, were included in multivariate Model 1. Model 2 was constructed as a more parsimonious model by prioritizing clinically meaningful variables. In this model, lesion pattern and lesion location were excluded to reduce the risk of overfitting, whereas treatment modality (procedure) was retained due to its significant association with baseline NIHSS and its potential confounding effect on early neurological outcomes.

Furthermore, to address the potential floor effect associated with low baseline NIHSS scores, we performed a sensitivity analysis restricted to patients with a baseline NIHSS >2. Because baseline NIHSS demonstrated a significant interaction with treatment modality, we also constructed two separate multivariable models to account for this relationship.

Subgroup analyses evaluated the FMD–ENI associations stratified by age, sex, stroke features, vascular risk factors, and cardiac parameters. Within each subgroup, logistic regression models were fitted to estimate the odds ratio (ORs) of FMD for ENI, and interaction effects were assessed by comparing the ORs between the subgroups. All statistical analyses were conducted using the IBM SPSS version 21.0 software (SPSS, Chicago, IL, USA). Statistical significance was set at *p* < 0.05.

## 3 Results

**Figure 1:**
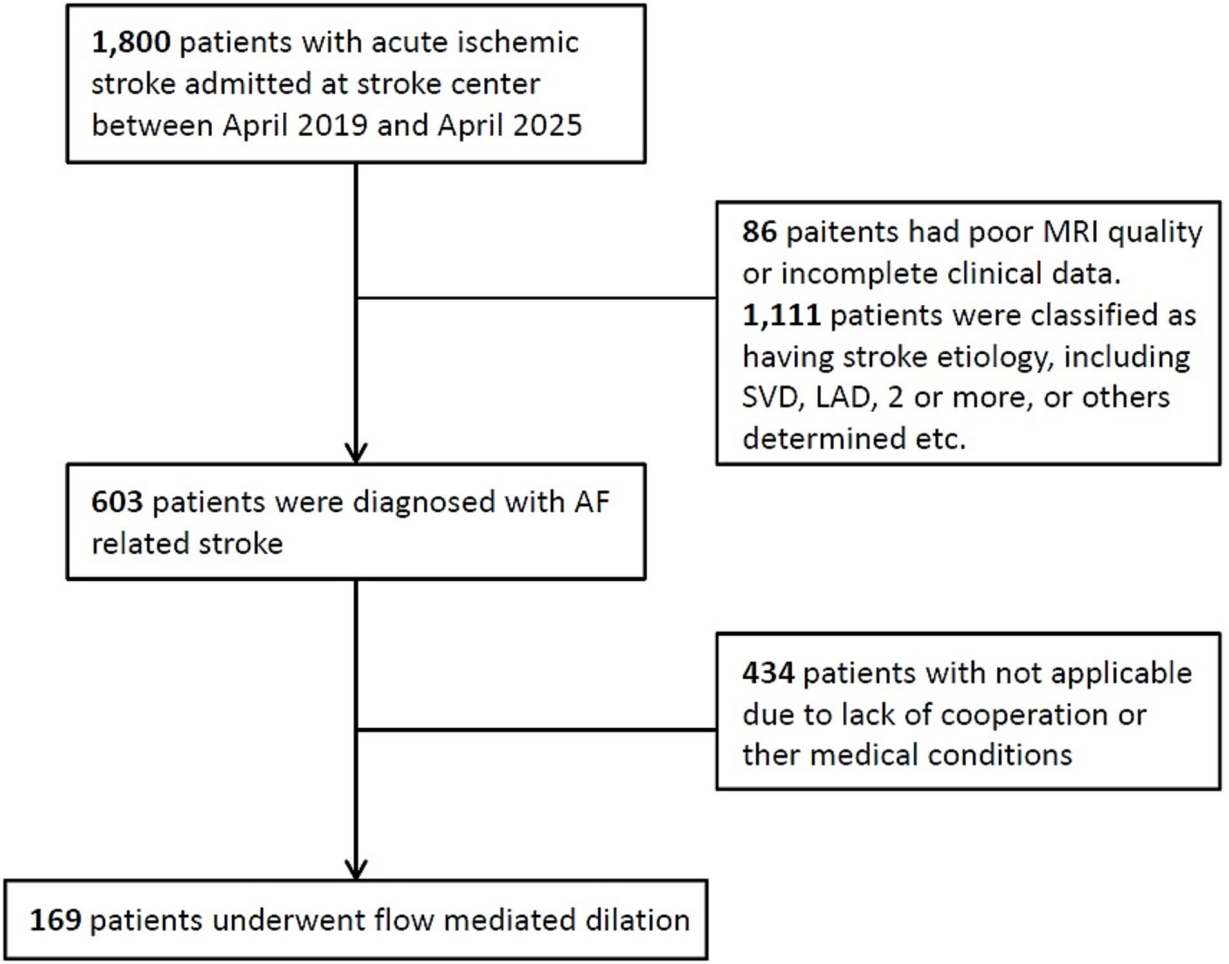
Study flow chart. AF, atrial fibrillation.

This study included 169 patients with acute AF-stroke who underwent FMD assessment (Figure 1). A total of 169 patients met inclusion criteria (mean age 72.5 ± 10.4 years, 100 [56.2%] male). FMD assessments were conducted within a median of 5 days (range, 3–6 days) from symptom onset. ENI occurred in 75 (44.4%) patients.

### 3.1 Baseline characteristics

The baseline characteristics stratified by ENI status are presented in Table 1. Those with ENI had higher NIHSS (7 [4–13] vs. 2 [1–5], *p* < 0.001), more confluent (38.7% vs. 25.5%) and scattered with confluent pattern (29.3% vs. 18.1%, *p* = 0.007), and higher %FMD (6.5% ± 2.5% vs. 5.3% ± 2.2%, *p* = 0.001).

**Table 1:** Baseline characteristics of patients with and without ENI.

	ENI− (*n* = 94)	ENI+ (*n* = 75)	*P*-value
Age (years)	73 ± 11	72 ± 10	0.762
Male	60 (63.8)	40 (53.3)	0.168
Hypertension	61 (64.9)	51 (68.0)	0.671
Diabetes mellitus	37 (39.4)	24 (32.0)	0.322
Hyperlipidemia	14 (14.9)	14 (18.7)	0.512
Current smoking history	14 (14.9)	22 (29.3)	0.023
Previous stroke history	15 (16.0)	10 (13.3)	0.633
Previous antithrombotic	27 (28.7)	22 (29.3)	0.931
Previous statin	28 (29.8)	22 (29.3)	0.949
Initial NIHSS score	2 (1–5)	7 (4–13)	< 0.001
Procedure			0.116
tPA	15 (16.0)	5 (6.7)	
EVT	8 (8.5)	7 (9.3)	
tPA with EVT	6 (6.4)	11 (14.7)	
Cardiac function			
Newly diagnosed AF	45 (47.9)	39 (52.0)	0.594
Ejection fraction, %	54 ± 15	55 ± 15	0.537
LAVI, mL/m^2^	60 ± 38	59 ± 27	0.260
Lesion patterns			0.007
Multiple scattered	53 (56.4)	24 (32.0)	
Single confluent	24 (25.5)	29 (38.7)	
Multiple with confluent	17 (18.1)	22 (29.3)	
Lesion location			0.152
Anterior	59 (62.8)	57 (76.0)	
Posterior	28 (29.8)	13 (17.3)	
Both	7 (7.4)	2 (6.7)	
FMD (%)	5.3 ± 2.2	6.5 ± 2.5	0.001

Results are presented as number (%), mean ± standard deviation, or interquartile range.

TOAST, Trial of Org 10172 in Acute Stroke Treatment; NIHSS, National Institutes of Health Stroke Scale; EVT, Endovascular thrombectomy; FMD, Flow-mediated dilatation.

Furthermore, patients in the 3rd tertile (highest %FMD > 6.30) were more female (69.6% vs. 64.0% vs. 46.0%; *p* = 0.023), had less history of previous statin use (32.1% vs. 42.0% vs. 17.5%; *p* = 0.016), had higher initial NIHSS score (3 [1–6] vs. 4 [1.5–7] vs. 5 [2–12]; *p* = 0.020), and had a higher proportion of ENI (35.7% vs. 38.0% vs. 57.1%; *p* = 0.035) (Supplementary Table 1).

### 3.2 Factors associated with ENI

Univariate analysis showed that the history of smoking, higher initial NIHSS score, confluent lesion and scattered with confluent lesion pattern, and higher FMD% were significantly associated with ENI.

In multivariate Model 1, which included variables with *p* < 0.1 from the univariate analysis along with clinically relevant covariates, current smoking (adjusted OR [aOR] = 4.532, 95% confidential interval [CI] 1.620–12.679; *p* = 0.004), higher initial NIHSS score (aOR = 1.329, 95% CI 1.195–1.477; *p* < 0.001), and higher FMD (%) (aOR = 1.179, 95% CI 1.005–1.382; *p* = 0.043) remained independently associated with ENI. In the multivariate Model 2, female sex (aOR = 0.472, 95% CI 0.227–0.982; *p* = 0.045), current smoking (aOR = 4.199, 95% CI 1.747–10.092; *p* = 0.001), and higher FMD (%) (aOR = 1.247, 95% CI 1.076–1.446; *p* = 0.003) were identified as independent factors associated with ENI (Table 2).

**Table 2:** Factors associated with ENI.

	Unadjusted univariate analysis		Adjusted multivariate analysis			
			Model 1^a^		Model 2^b^	
	OR (95% CI)	*P*-value	aOR (95% CI)	*P*-value	aOR (95% CI)	*P*-value
Age (years)	0.996 (0.969–1.023)	0.749	–			
Male	0.648 (0.349–1.202)	0.169	–		0.472 (0.227–0.982)	0.045
Hypertension	1.150 (0.604–2.189)	0.671				
Diabetes mellitus	0.725 (0.383–1.372)	0.323				
Hyperlipidemia	1.311 (0.582–2.955)	0.513				
Current smoking history	2.372 (1.115–5.045)	0.025	4.532 (1.620–12.679)	0.004	4.199 (1.747–10.092)	0.001
Previous stroke history	0.810 (0.341–1.924)	0.633				
Previous antithrombotic	1.030 (0.528–2.010)	0.931				
Previous statin	0.978 (0.503–1.903)	0.949				
Initial NIHSS score	1.336 (1.208–1.476)	< 0.001	1.329 (1.195–1.477)	< 0.001		
Procedure						
None	1 (Reference)					
tPA	0.417 (0.142–1.222)	0.111				
EVT	1.094 (0.372–3.214)	0.871				
tPA with EVT	2.292 (0.794–6.611)	0.125				
Cardiac function						
Newly diagnosed AF	1.180 (0.643–2.165)	0.594				
Ejection fraction, %	1.008 (0.986–1.032)	0.479				
LAVI, mL/m^2^	1.000 (0.989–1.010)	0.941				
Lesion patterns						
Multiple scattered	1 (Reference)		–			
Single confluent	2.668 (1.293–5.507)	0.008				
Multiple with confluent	2.858 (1.290–6.333)	0.010				
Lesion location						
Anterior	1 (Reference)					
Posterior	0.481 (0.227–1.019)	0.056				
Both	0.739 (0.222–2.465)	0.623				
FMD (%)	1.237 (1.076–1.423)	0.003	1.179 (1.005–1.382)	0.043	1.247 (1.076–1.446)	0.003

The results are presented as odds ratios (ORs) and 95% confidence interval (95% CI).

NIHSS, National Institutes of Health Stroke Scale; tPA, tissue plasminogen activator; EVT, endovascular thrombectomy; FMD, Flow-mediated dilation; EF, ejection fraction; LAVI, left atrial volume index; AF, atrial fibrillation.

a Adjusted for age, sex (male), smoking, initial NIHSS, lesion pattern, location, and FMD.

b Adjusted for age, sex (male), smoking, procedure, and FMD.

A sensitivity analysis restricted to patients with a baseline NIHSS >2 showed that a higher %FMD remained significantly associated with ENI (Supplementary Tables 2, 3).

### 3.3 Subgroup analysis

**Figure 2:**
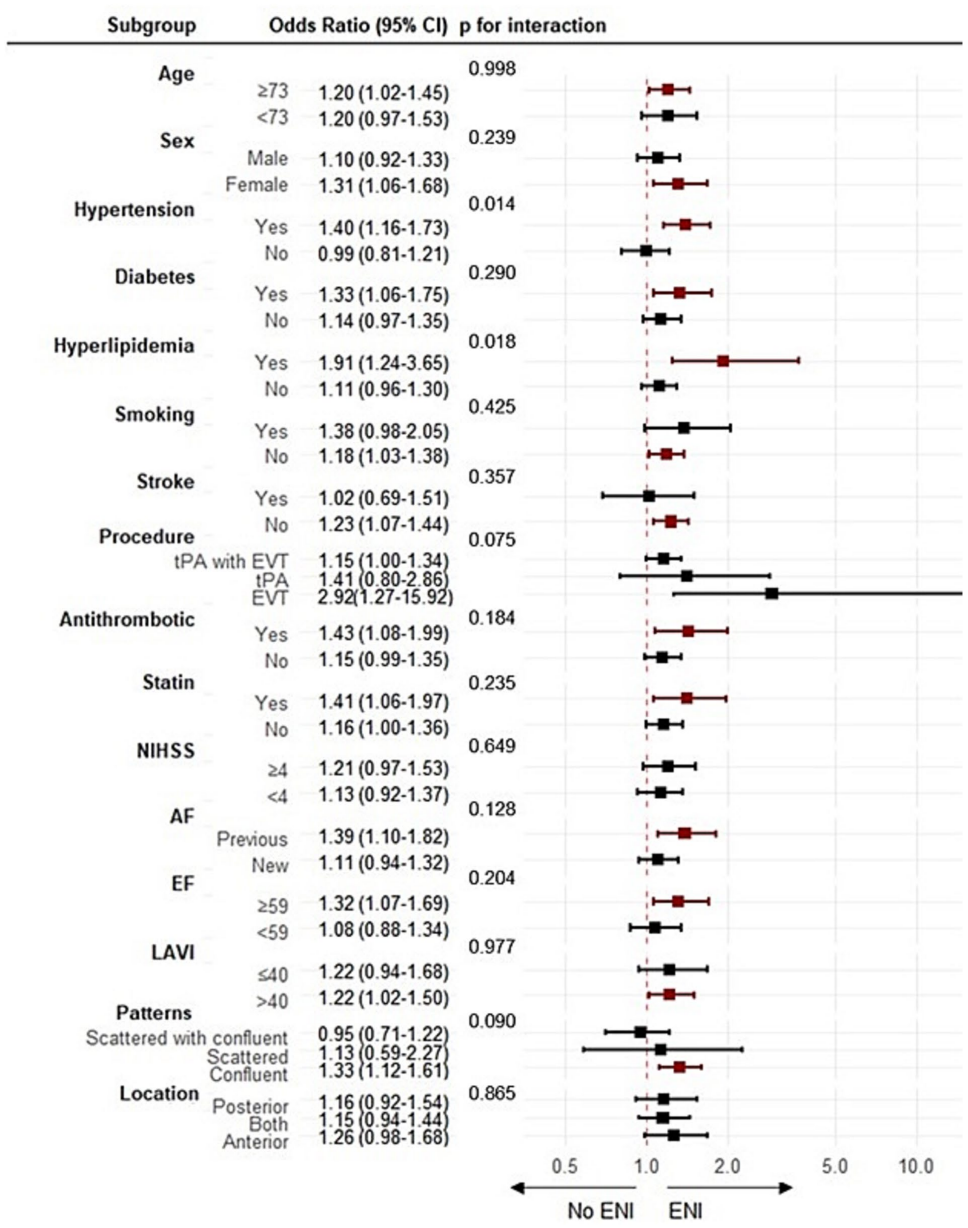
Subgroup analysis. Logistic analysis of the association between endothelial function and early neurological improvement. CI, confidential interval; tPA, tissue plasminogen activator; EVT, endovascular thrombectomy; NIHSS, National Institutes of Health Stroke Scale; AF, atrial fibrillation; EF, ejection fraction; LAVI, left atrial volume index; ENI, early neurological improvement. The red squares and corresponding 95% CI lines represent the odds ratios for subgroups where the *p*-value is less than 0.05.

Subgroup analyses were performed to examine whether the treatment effects of FMD differed across clinically relevant patient subgroups. Statistically significant interactions were identified for hypertension (interaction OR: 1.41, 95% CI: 1.07–1.89, *p* = 0.016) and hyperlipidemia (interaction OR: 1.72, 95% CI: 1.08–3.33, *p* = 0.018). In contrast, no significant interaction effects were observed in the other subgroups (Figure 2).

## 4 Discussion

In the present study, ENI was observed in 44.4% of the patients with AF-stroke. We identified a higher NIHSS score, smoking history, and higher %FMD as independent factors associated with ENI in patients with AF-stroke, especially the presence of concomitant vascular risk factors, such as hypertension and dyslipidemia.

AF is closely associated with endothelial dysfunction. Irregular and turbulent blood flow in AF reduces shear stress, impairs nitric oxide (NO) production, and promotes endothelial injury (20). Elevated levels of asymmetric dimethylarginine and soluble CD40 ligand, which are biomarkers of endothelial dysfunction, have also been observed in AF compared with sinus rhythm (20). Furthermore, endothelial dysfunction, in turn, contributes to atrial remodeling, thrombogenesis, and hemodynamic alterations that facilitate AF and AF-stroke (13, 14). These patients are often prone to hemorrhagic transformation due to increased BBB permeability (1) and typically present with neurological severity and larger infarcts (2, 4, 5) Nevertheless, ENI can still occur in a subset of patients with AF-stroke and is associated with favorable functional outcomes (6).

We demonstrated that higher %FMD was associated with the occurrence of ENI in patients with AF-stroke. Several mechanisms can explain this association. First, preserved endothelial function may sustain adequate cerebral perfusion through vasoactive mediators, such as NO and endothelium-derived hyperpolarizing factor, thereby supporting tissue viability (11). Second, preserved endothelial progenitor cell activity and reduced matrix metalloproteinase-9 (MMP-9) activation help maintain BBB integrity (21). Beyond vascular regulation, endothelial-derived NO exerts direct neuroprotective and anti-inflammatory effects that may enhance neuronal survival, synaptic plasticity, and overall neural recovery after ischemia (22). Notably, a higher %FMD was more strongly associated with ENI in patients exhibiting typical cardioembolic features, including known AF, left atrial enlargement, and confluent lesions or higher NIHSS. Although these interactions were not statistically significant, this pattern may reflect the greater dependence of cardioembolic strokes on endothelial-related vascular reserve, suggesting a protective role of intact endothelium in recovery after AF-stroke.

We also found that current smoking history was associated with ENI in patients with AF-stroke. While active smoking is well-known to impair endothelial function and worsen stroke outcomes, the positive association of smoking with early outcome, known as the “smoker’s paradox,” might be attributed to mechanisms such as ischemic preconditioning or enhanced fibrinolytic activity in smokers (23, 24). However, this association may indeed be influenced by residual confounding or unmeasured variables. Furthermore, we found a stronger correlation between higher %FMD and ENI in patients with concomitant vascular risks, such as hypertension or dyslipidemia. Patients with AF often have multiple vascular comorbidities, and previous studies have shown that patients with concurrent hypertension exhibit poorer endothelial function than those with AF alone (25). Under such conditions, preserved endothelial function may reflect greater vascular stability and adaptive capacity to ischemic stress, facilitating improved collateral recruitment and the maintenance of BBB integrity (26). Consequently, these patients may experience enhanced reperfusion and faster neurological recovery following cerebral ischemia (27, 28).

This study has some limitations. First, it was conducted at a single center with a retrospective design, which may have missed potential ENI variables, such as recanalization status. Given the limited sample size within each revascularization subgroup, the potential differential mechanisms linking FMD to ENI could not be fully evaluated in this study. Further large-scale investigations are warranted to confirm these findings (Supplementary Figure 1). Second, unlike the 2025 study which provided a general overview of stroke subtypes (12), this work performs a deep dive into AF-specific pathophysiology. We have incorporated detailed echocardiographic parameters, such as LAVI and LVEF, alongside specific embolic lesion patterns, but other cardiac markers (e.g., E/e ratio, BNP, atrial strain, or diastolic indices) were not assessed. Therefore, our findings may underestimate the role of cardiac remodeling and hemodynamic compromise in ENI. Third, although FMD was initially intended to serve as a predictor of ENI, most measurements were performed several days after admission, with a median timing of 5 days from stroke onset, and therefore after ENI assessment. While previous studies suggest that endothelial function, as assessed by FMD, remains relatively stable during the acute phase of ischemic stroke (28, 29), the possibility of reverse causality cannot be fully excluded in the present retrospective design. Specifically, ENI itself may have influenced endothelial function at the time of measurement. Accordingly, the observed association between higher %FMD and ENI should be interpreted with caution. Finally, a limitation of this study is the potential for selection bias. Patients who did not undergo FMD measurement were significantly older and presented with more severe strokes at baseline (Supplementary Table 5). Consequently, the generalizability of our findings to the very elderly or those with severe initial neurological impairment may be limited.

Despite these limitations, our findings demonstrate that a higher %FMD is independently associated with ENI in AF-stroke. This association underscores the potential contribution of endothelial function to early neurological recovery. While further research is required, these results suggest that FMD may serve as a meaningful biomarker in AF-stroke and provide a valuable foundation for future prospective validation of its utility in risk stratification and prognostic assessment.

## Data Availability

The raw data supporting the conclusions of this article will be made available by the authors, without undue reservation.
