# Supplementary material for: Association between endothelial function and early neurological improvement in atrial fibrillation-related ischemic stroke
Source: Front Neurol. 2026 Jan 29;17:1733034. doi: 10.3389/fneur.2026.1733034 (PMC12894233; doi:10.3389/fneur.2026.1733034)
Supplement: Supplementary file 1 [file Table_1.DOCX]

Supplementary Material

**Supplementary Table 1. Baseline characteristics of patients with AF-related stroke according to the terciles of the FMD %**

|  | **FMD %** | | | |
| --- | --- | --- | --- | --- |
|  | < 4.90 | 4.90–6.30 | 6.30 < | P-value |
| Age (years) | 69 ± 12 | 73 ± 11 | 74 ± 10 | 0.093 |
| Male | 39 (69.6) | 32 (64.0) | 29 (46.0) | 0.023 |
| Hypertension | 36 (64.3) | 35 (70.0) | 41 (65.1) | 0.799 |
| Diabetes mellitus | 21 (37.5) | 19 (38.0) | 21 (33.3) | 0.846 |
| Hyperlipidemia | 10 (17.9) | 8 (16.0) | 10 (15.9) | 0.951 |
| Smoking history | 16 (28.6) | 8 (16.0) | 12 (19.0) | 0.247 |
| Previous stroke history | 9 (16.1) | 9 (18.0) | 7 (11.1) | 0.560 |
| Previous antithrombotic | 18 (32.1) | 15 (30.0) | 16 (25.4) | 0.708 |
| Previous statin | 18 (32.1) | 21 (42.0) | 11 (17.5) | 0.016 |
| Initial NIHSS score | 3 (1–6) | 4 (1.5–7) | 5 (2–12) | 0.020 |
| **Procedure** |  |  |  | 0.385 |
| tPA | 5 (8.9) | 6 (12.0) | 9 (14.3) |  |
| EVT | 4 (7.1) | 6 (12.0) | 5 (7.9) |  |
| tPA with EVT | 5 (8.9) | 2 (4.0) | 10 (15.9) |  |
| **Cardiac function** |  |  |  |  |
| Newly diagnosed AF | 29 (51.8) | 19 (38.0) | 36 (57.1) | 0.121 |
| Ejection fraction, % | 54 ± 14 | 56 ± 13 | 54 ± 16 | 0.817 |
| LAVI, ml/m^2^ | 35 ± 5 | 19 ± 3 | 38 ± 5 | 0.815 |
| **Lesion patterns** |  |  |  | 0.900 |
| Scattered | 27 (48.2) | 23 (46.0) | 27 (42.9) |  |
| Confluent | 15 (26.8) | 17 (34.0) | 21 (33.3) |  |
| Scattered with confluent | 14 (25.0) | 10 (20.0) | 15 (23.8) |  |
| **Lesion location** |  |  |  | 0.259 |
| Anterior | 38 (67.9) | 30 (60.0) | 48 (76.2) |  |
| Posterior | 12 (21.4) | 16 (32.0) | 13(20.6) |  |
| Both | 6 (10.7) | 4 (8.0) | 2 (3.2) |  |
| **ENI** | 20 (35.7) | 19 (38.0) | 36 (57.1) | 0.035 |

The results are presented as numbers and percentages or as means ± standard deviations or median (interquartile ranges).

**Supplementary table 2. Baseline characteristics of patients with and without ENI**

|  | **ENI-**  **(n = 89)** | **ENI +**  **(n = 75)** | ***P*-value** |
| --- | --- | --- | --- |
| Age (years) | 73 ± 11 | 72 ± 10 | 0.752 |
| Male | 57 (64.0) | 40 (53.3) | 0.164 |
| Hypertension | 58 (65.2) | 51 (68.0) | 0.702 |
| Diabetes mellitus | 36 (40.4) | 24 (32.0) | 0.263 |
| Hyperlipidemia | 13 (14.6) | 14 (18.7) | 0.485 |
| Smoking history | 12 (13.5) | 22 (29.3) | 0.013 |
| Previous stroke history | 14 (15.7) | 10 (13.3) | 0.665 |
| Previous antithrombotic | 26 (29.2) | 22 (29.3) | 0.987 |
| Previous statin | 27 (30.3) | 22 (29.3) | 0.889 |
| Initial NIHSS score | 4 (3 – 5) | 7 (4 – 13) | <0.001 |
| **Procedure** |  |  | 0.114 |
| Tpa | 15 (16.9) | 5 (6.7) |  |
| EVT | 8 (9.0) | 7 (9.3) |  |
| tPA with EVT | 6 (6.7) | 11 (14.7) |  |
| **Cardiac function** |  |  |  |
| Newly diagnosed AF | 44 (49.4) | 39 (52.0) | 0.744 |
| Ejection fraction, % | 54 ± 14 | 55 ± 15 | 0.617 |
| LAVI, mL/m^2^ | 60 ± 38 | 59 ± 27 | 0.314 |
| **Lesion patterns** |  |  | 0.013 |
| Multiple scattered | 49 (55.1) | 24 (32.0) |  |
| Single Confluent | 23 (25.8) | 29 (38.7) |  |
| Multiple with confluent | 17 (19.1) | 22 (29.3) |  |
| **Lesion location** |  |  | 0.155 |
| Anterior | 57 (64.0) | 57 (76.0) |  |
| Posterior | 27 (30.3) | 13 (17.3) |  |
| Both | 5 (5.6) | 5 (6.7) |  |
| **FMD (%)** | 5.3 ± 2.2 | 6.5 ± 2.5 | 0.001 |

Results are presented as number (%), mean ± standard deviation, or interquartile range.

TOAST; Trial of Org 10172 in Acute Stroke Treatment, NIHSS; National Institutes of Health Stroke Scale, EVT; Endovascular thrombectomy, FMD: Flow-mediated dilatation.

**Supplementary table 3. Factors associated with ENI**

|  | **Unadjusted univariate analysis** | | **Adjusted multivariate analysis** | | | |
| --- | --- | --- | --- | --- | --- | --- |
|  |  | | **Model 1^*^** | | **Model 2^#^** | |
|  | **OR (95% CI)** | ***P*-value** | **aOR (95% CI)** | ***P*-value** | **aOR (95% CI)** | ***P*-value** |
| Age (years) | 0.995 (0.967 – 1.023) | 0.702 | - |  |  |  |
| Male | 0.642 (0.343 – 1.201) | 0.165 | 0.466 (0.201 – 1.082) | 0.076 | 0.443 (0.208 – 0.943) | 0.035 |
| Hypertension | 1.136 (0.592 – 2.181) | 0.702 |  |  |  |  |
| Diabetes mellitus | 0.693 (0.364 – 1.319) | 0.264 |  |  |  |  |
| Hyperlipidemia | 1.342 (0.587 – 3.067) | 0.486 |  |  |  |  |
| Current smoking history | 2.664 (1.214 – 5.843) | 0.015 | 4.424 (1.614 – 12.123) | 0.004 | 5.216 (2.007 – 13.555) | 0.001 |
| Previous stroke history | 0.824 (0.343 – 1.981) | 0.666 |  |  |  |  |
| Previous antithrombotic | 1.006 (0.512 – 1.976) | 0.987 |  |  |  |  |
| Previous statin | 0.953 (0.487 – 1.866) | 0.889 |  |  |  |  |
| Initial NIHSS score | 1.343 (1.206 – 1.495) | <0.001 | 1.330 (1.188 – 1.489) | < 0.001 |  |  |
| Procedure |  |  |  |  |  |  |
| None | 1 (reference) |  |  |  | 1 (reference) |  |
| tPA | 0.385 (0.131 – 1.130) | 0.082 |  |  | 0.289 (0.092 – 0.906) | 0.033 |
| EVT | 1.010 (0.343 – 2.974) | 0.986 |  |  | 1.259 (0.392 – 4.041) | 0.699 |
| tPA with EVT | 2.115 (0.732 – 6.117) | 0.167 |  |  | 1.523 (0.488 – 4.749) | 0.469 |
| Cardiac function |  |  |  |  |  |  |
| Newly diagnosed AF | 1.108 (0.599 – 2.049) | 0.744 |  |  |  |  |
| Ejection fraction, % | 1.006 (0.983 – 1.030) | 0.610 |  |  |  |  |
| LAVI, mL/m^2^ | 0.999 (0.988 – 1.010) | 0.853 |  |  |  |  |
| Lesion patterns |  |  |  |  |  |  |
| Multiple scattered | 1 (reference) |  | - |  |  |  |
| Single Confluent | 2.574 (1.236 – 5.360) | 0.012 |  |  |  |  |
| Multiple with confluent | 2.642 (1.188 – 5.877) | 0.017 |  |  |  |  |
| Lesion location |  |  | - |  |  |  |
| Anterior | 1 (reference) |  |  |  |  |  |
| Posterior | 0.481 (0.226 – 1.026) | 0.058 |  |  |  |  |
| Both | 1.000 (0.274 – 3.643) | >0.999 |  |  |  |  |
| FMD (%) | 1.231 (1.069 – 1.417) | 0.004 | 1.171 (1.001 – 1.371) | 0.049 | 1.252 (1.072 – 1.463) | 0.005 |

The results are presented as odds ratios (ORs) and 95% confidence interval (95% CI).

NIHSS: National Institutes of Health Stroke Scale, tPA; tissue plasminogen activator, EVT; endovascular thrombectomy, FMD: Flow-mediated dilation, EF; ejection fraction, LAVI; left atrial volume index, AF; atrial fibrillation

^*^ Adjusted for age, sex (male), smoking, initial NIHSS, lesion pattern, location, and FMD

^#^ Adjusted for age, sex (male), smoking, procedure, and FMD

**Supplementary table 4. Group differences**

|  | **ENI-**  **(n = 94)** | **ENI +**  **(n = 75)** | ***P*-value** | ***Effect size or standard deviation*** |
| --- | --- | --- | --- | --- |
| Age (years) | 73 ± 11 | 72 ± 10 | 0.762 | Cohen's d = 0.05 |
| Male | 60 (63.8) | 40 (53.3) | 0.168 | OR = 0.65 |
| Hypertension | 61 (64.9) | 51 (68.0) | 0.671 | OR = 1.15 |
| Diabetes mellitus | 37 (39.4) | 24 (32.0) | 0.322 | OR = 0.73 |
| Hyperlipidemia | 14 (14.9) | 14 (18.7) | 0.512 | OR = 1.31 |
| Smoking history | 14 (14.9) | 22 (29.3) | 0.023 | OR = 2.36 |
| Current stroke history | 15 (16.0) | 10 (13.3) | 0.633 | OR = 0.81 |
| Previous antithrombotic | 27 (28.7) | 22 (29.3) | 0.931 | OR = 1.03 |
| Previous statin | 28 (29.8) | 22 (29.3) | 0.949 | OR = 0.98 |
| Initial NIHSS score | 2 (1–5) | 7 (4–13) | < 0.001 | Cohen's d = -1.23 |
| **Procedure** |  |  | 0.116 |  |
| tPA | 15 (16.0) | 5 (6.7) |  | -0.297 |
| EVT | 8 (8.5) | 7 (9.3) |  | 0.029 |
| tPA with EVT | 6 (6.4) | 11 (14.7) |  | 0.272 |
| **Cardiac function** |  |  |  |  |
| Newly diagnosed AF | 45 (47.9) | 39 (52.0) | 0.594 | OR = 1.18 |
| Ejection fraction, % | 54 ± 15 | 55 ± 15 | 0.537 | Cohen's d = -0.12 |
| LAVI, mL/m^2^ | 60 ± 38 | 59 ± 27 | 0.260 | Cohen's d = 0.01 |
| **Lesion patterns** |  |  | 0.007 |  |
| Multiple scattered | 53 (56.4) | 24 (32.0) |  | -0.506 |
| Single Confluent | 24 (25.5) | 29 (38.7) |  | 0.284 |
| Multiple with confluent | 17 (18.1) | 22 (29.3) |  | 0.267 |
| **Lesion location** |  |  | 0.152 |  |
| Anterior | 59 (62.8) | 57 (76.0) |  | 0.290 |
| Posterior | 28 (29.8) | 13 (17.3) |  | -0.297 |
| Both | 7 (7.4) | 2 (6.7) |  | -0.03 |
| **FMD (%)** | 5.3 ± 2.2 | 6.5 ± 2.5 | 0.001 | Cohen's d = -0.49 |

**Supplementary Table 5.** Baseline Characteristics of Patients With and without FMD measurements

|  | **FMD +**  **n = 169** | **FMD -**  **n = 434** | **p-value** |
| --- | --- | --- | --- |
| Age (years) | 72 ± 11 | 76 ± 10 | <0.001 |
| Male sex | 100 (59.2) | 231 (53.2) | 0.188 |
| Initial NIHSS | 4 (2 – 8) | 7 (2 – 14) | <0.001 |

Results are presented as number and percent (% column) or mean ± SD or median [IQR]. IQR, interquartile range; NIHSS, National Institutes of Health Stroke Scale; SD, standard deviation

**Supplementary figure 1. Association between FMD and ENI depending on the procedure**

**
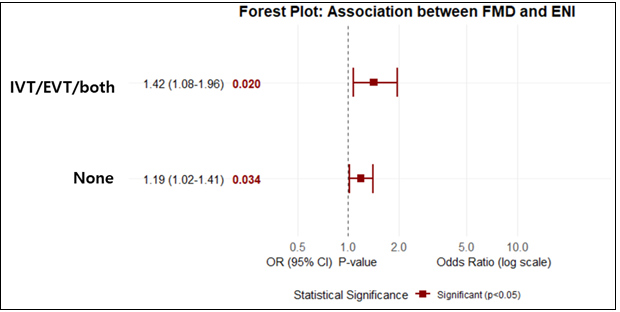
**

EVT, endovascular therapy; IVT, Intravenous thrombolysis; FMD, flow mediated dilation; ENI, early neurological improvement
